# Actual Hydrated Volume of Acellular Dermal Matrix in Prepectoral Breast Reconstruction

**DOI:** 10.3390/jcm15155948

**Published:** 2026-07-30

**Authors:** In-Woo Chu, Jun-Ho Lee

**Affiliations:** Department of Plastic and Reconstructive Surgery, Yeungnam University College of Medicine, Daegu 42415, Republic of Korea; 22556067@yu.ac.kr

**Keywords:** acellular dermal matrix, prepectoral breast reconstruction, hydrated volume, implant-based breast reconstruction, calculated thickness, volumetric planning

## Abstract

**Background/Objectives**: Acellular dermal matrix (ADM) is increasingly used in prepectoral breast reconstruction, but operative planning still relies largely on label-reported size and thickness. In this study, we evaluated the actual post-hydration volumetric properties of commercially available ADMs and developed manufacturer-specific reference estimates for clinical use. **Methods**: In this single-center retrospective study, we analyzed 200 ADM sheets used in 173 patients between February 2023 and July 2025. Standardized 20 × 20 cm^2^ sheets from four manufacturers were hydrated, their true volumes were measured using the water displacement method, their measured hydrated areas were recorded, and their calculated thicknesses were derived from the hydrated volume divided by the measured hydrated area. Differences based on manufacturer and thickness group were analyzed, and manufacturer-specific reference estimates for other sizes were derived and compared with independent 18 × 18 cm^2^ validation data. **Results**: In all groups, the measured hydrated volume and measured hydrate area exceeded label-reported expectations. The measured hydrated volume and calculated thickness differed significantly according to the manufacturer and thickness group, and the interaction between these factors was also significant. The measured hydrated area varied less across thickness groups, although inter-manufacturer differences were observed within the shared 1.0–2.0 mm category. Validation with independent 18 × 18 cm^2^ data showed overall agreement with the derived reference estimates. **Conclusions**: Post-hydration ADM volumetric properties differ according to manufacturer and thickness group, largely because of differences in calculated thickness rather than area alone. The proposed manufacturer-specific reference table may serve as a practical aid for volumetric planning in prepectoral breast reconstruction.

## 1. Introduction

Implant-based breast reconstruction (IBBR) following mastectomy has been widely adopted because of its shorter operative time, technical simplicity, and faster postoperative recovery [1]. In recent years, the introduction of acellular dermal matrix (ADM) has enabled broader implant coverage [2,3,4,5], contributing to a shift from subpectoral to prepectoral reconstruction and leading to a renewed interest in IBBR [6,7,8,9]. In prepectoral reconstruction, ADM plays a critical role in broad implant coverage, pocket formation, soft-tissue support, and the maintenance of shape stability [6,10,11]. As its clinical application has expanded, ADM has been used in varied configurations, including total wrapping and anterior wrapping techniques [10,12,13]. Accordingly, the amount and physical properties of ADM used in practice are now directly related to reconstructive outcomes [14,15]. As ADM occupies an increasing proportion of the total reconstructed breast volume, its volumetric contribution has become an increasingly important consideration [13,16].

Despite this growing importance, ADM volume in clinical practice is still often estimated based on label-reported size and thickness. However, this approach is inherently limited because ADM is used intraoperatively in a hydrated rather than a dry state, and its measured hydrated area and volume may not correspond to the labeled values. In particular, previous studies have suggested that ADM thickness and area may influence clinical outcomes, drainage volume, complications, and final symmetry [14,15,17]; however, quantitative data comparing the actual post-hydration volumetric characteristics of different commercially available ADMs remain insufficient. In our previous study, we used three-dimensional breast scanning (Vectra) to precisely analyze soft-tissue and volumetric changes before and after breast reconstruction [18]; in the process, we identified the need for a quantitative reference standard for the actual volume occupied by ADM to improve analytical accuracy. Among the standardized methods available for measuring the true volume of ADM, the water displacement method is considered the most accurate. Based on hydrodynamic principles, this technique defines ADM volume as the amount of displaced water, thereby allowing the precise measurement of the true volume of ADM in a hydrated state.

The purposes of this study were twofold: First, using 20 × 20 cm^2^ ADMs actually used in prepectoral breast reconstruction, we measured and compared true post-hydration volume, surface area, and post-hydration thickness among manufacturers and thickness groups. Post-hydration thickness was defined as the calculated thickness derived from the volume per unit area (cc/cm^2^) and converted into a linear thickness value in millimeters or centimeters when appropriate. Second, based on manufacturer- and thickness-group-specific calculated thickness values obtained from these measurements, we estimated hydrated ADM volume for other size–thickness combinations and developed a manufacturer-specific reference table applicable in actual surgical planning. Accordingly, the reference table presented in this study may serve as a more realistic volumetric planning reference for prepectoral breast reconstruction.

## 2. Materials and Methods

### 2.1. Study Design and Patients

In this single-center retrospective study, we measured the hydrated volume, hydrated area, and calculated thickness values of 20 × 20 cm^2^ acellular dermal matrix (ADM) samples according to manufacturer and thickness group; then, based on these data, we derived a manufacturer-specific size–thickness reference table. The reference table was constructed based on official statements or documentation from the manufacturers confirming that products from the same manufacturer were produced through the same manufacturing process and that differences in size were attributable solely to trimming.

A total of 200 ADM samples used in 173 patients who underwent prepectoral breast reconstruction at our institution between 1 February 2023, and 15 July 2025, were analyzed.

The ADM manufacturers and specifications were as follows: CGDerm^®^ (CGBio, Seoul, Republic of Korea), MegaDerm^®^ (L&C Bio Co., Ltd., Seoul, Republic of Korea), MyDerm^®^ (MSBIO, Inc., Seongnam, Gyeonggi, Republic of Korea), and SCDerm^®^ (DOF Inc., Hwaseong, Gyeonggi, Republic of Korea). All analyzed ADMs measured 20 × 20 cm^2^. The thickness categories for direct measurement were 1.0–1.5 mm, 1.0–2.0 mm, 1.5–1.9 mm, and 1.9–2.3 mm, whereas the derived reference table also included a 2.0–3.0 mm category. The sample composition was as follows: 35 CGDerm^®^ samples of 1.0–2.0 mm, 45 MegaDerm^®^ samples of 1.0–1.5 mm, 24 MegaDerm^®^ samples of 1.5–1.9 mm, 18 MegaDerm^®^ samples of 1.9–2.3 mm, 48 MyDerm^®^ samples of 1.0–2.0 mm, and 30 SCDerm^®^ samples of 1.0–2.0 mm (Table 1).

All analyzed sheets were labeled as 20 × 20 cm and used in prepectoral implant-based breast reconstruction. The manufacturers were CGDerm^®^ (CGBio, Seoul, Republic of Korea), MegaDerm^®^ (L&C Bio Co., Ltd., Seoul, Republic of Korea), MyDerm^®^ (MSBIO, Inc., Seongnam, Gyeonggi, Republic of Korea), and SCDerm^®^ (DOF Inc., Hwaseong, Gyeonggi, Republic of Korea). Thickness ranges reflected manufacturer-reported categories. N indicates the number of ADM sheets analyzed per manufacturer and thickness subgroup, and Total (%) indicates the contribution of each subgroup to the overall sample (N = 200).

### 2.2. Measurement of Hydrated ADM Volume and Area

The ADM samples underwent a standardized hydration process, after which the hydrated volume was determined using the fluid displacement method. Each matrix was fully submerged in room-temperature normal saline (maintained between 18 °C and 24 °C depending on the operating room conditions) within a cylindrical titanium vessel. The vessel had a total capacity of 800 mL to comfortably accommodate all ADM dimensions. To ensure complete removal of residual manufacturing or preservative substances, the ADM was gently agitated using a titanium wide spatula. Notably, the required agitation duration varied among individual products (ranging from less than 30 s to over 2 min) due to inherent, batch-to-batch variations in surface substance density arising from the manufacturing process.

Following thorough rinsing, the matrix was gently blotted and compressed until no water droplets formed. This endpoint was determined by a single, highly experienced operator to ensure structural consistency and prevent material damage. Because prioritizing the preservation of optimal matrix elasticity was critical over mechanical stretching, the structural and volumetric assessment was performed only a single time for each sample. Furthermore, this assessment was strictly limited to the pre-implantation phase, as the matrix becomes permanently unavailable for ex vivo calculation once it is inset to cover the breast implant.

A measured displacement of 1 cc was considered equivalent to an actual ADM volume of 1 cc. The hydrated length and width of each sample were also recorded, and the hydrated area (cm^2^) was calculated accordingly. In this study, the measured hydrated volume referred not to the theoretical volume of ADM in the dry state but to the effective volume in the hydrated state, which more closely reflects the condition in which ADM was used intraoperatively.

### 2.3. Definition of Derived Variables

The calculated thickness of ADM for each manufacturer and thickness group was defined as the ratio of measured hydrated volume to measured hydrated area:

Calculated thickness (cc/cm^2^ or cm) = measured hydrated volume (cc)/measured hydrated area (cm^2^).

Because this parameter is dimensionally equivalent to length, it was interpreted as thickness in centimeters and converted to millimeters when appropriate.

### 2.4. Derivation of Size- and Thickness-Specific Estimated Volume (Reference Estimate)

Because hydrated volume was not directly measured for ADMs of all available sizes, the mean calculated thickness and standard deviation for each manufacturer and thickness subgroup were first obtained from the directly measurable 20 × 20 cm^2^ samples. Thereafter, under the assumption that the post-hydration volumetric property per unit area remains relatively constant within the same manufacturer and thickness subgroup, the estimated hydrated volume for other sizes was calculated as follows:

Estimated hydrated volume (cc) = targeted area (cm^2^) × subgroup mean calculated thickness (cm).

The standard deviation was calculated under the assumption that the variation in calculated thickness was linearly propagated in proportion to area. In addition, the 95% confidence interval (CI) for the mean estimated value and the 95% prediction interval (PI) for a single future sheet were calculated. Accordingly, the size–thickness reference table presented in this study should be interpreted not as a set of direct measurements but as a series of derived estimates generated from actually measured subgroup parameters.

### 2.5. Statistical Analysis

Within each manufacturer and thickness subgroup, a one-sample t-test was performed to compare the actually measured values with the estimated values. Continuous variables were presented as the mean, standard deviation, median, interquartile range, minimum, maximum, and 95% confidence interval. Normality within each subgroup was assessed using the Shapiro–Wilk test. Differences in measured hydrated volume, measured hydrated area, and calculated thickness according to thickness group were analyzed using one-way analysis of variance (ANOVA) and two-way ANOVA. In the shared thickness category of 1.0–2.0 mm, additional between-manufacturer comparisons were performed separately. Statistical significance was defined as *p* < 0.05.

## 3. Results

### 3.1. Measured Hydrated ADM Volume

**Figure 1:**
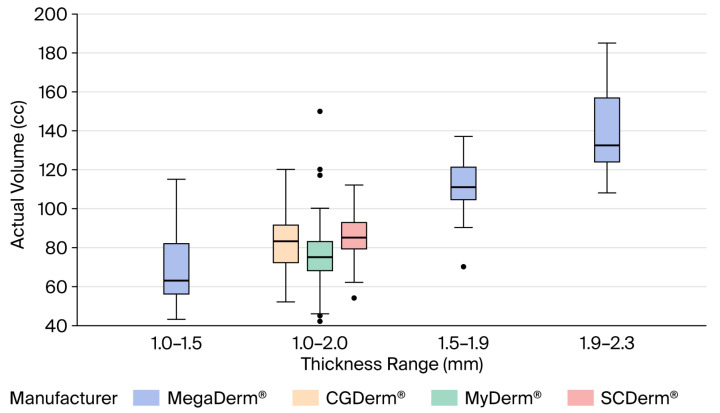
Measured hydrated ADM volumes categorized by manufacturer and thickness range.

The estimated hydrated volume and the measured hydrated volume differed significantly between manufacturers, with all groups showing higher mean hydrated volumes than the estimated hydrated volumes (SCDerm^®^, 85.87 cc; MyDerm^®^, 76.75 cc; CGDerm^®^, 84.63 cc; and MegaDerm^®^, 68.20 cc, 111.5 cc, and 140.67 cc, according to thickness subgroup) (Table 2). The measured hydrated volume also differed markedly according to the manufacturer and thickness group (Table 3, Figure 1). For two-way ANOVA, both the manufacturer (F = 33.67, *p* < 0.001) and thickness group (F = 123.58, *p* < 0.001) showed statistically significant effects. In addition, the interaction between manufacturer and thickness group was significant (F = 60.51, *p* < 0.001) (Supplementary Table S2).

The mean estimated hydrated volume was calculated from the label-reported area (400 cm^2^) multiplied by the midpoint of the label-reported thickness range converted to centimeters. Measured hydrated volume was obtained after standardized hydration using the water displacement method, in which 1 cc of displaced water was considered equivalent to 1 cc of ADM. Values are presented as mean ± SD. Mean difference represents the measured hydrated volume minus the estimated hydrated volume. *p*-values were obtained using one-sample t tests comparing the measured hydrated volume with the mean estimated hydrated volume within each manufacturer and thickness subgroup.

The measured hydrated volume (cc) was recorded for each ADM sheet after standardized hydration using the water displacement method. For each manufacturer and thickness subgroup, the table reports the mean, SD, and Shapiro–Wilk test *p*-values for normality, the minimum, the 25th percentile, the median, the 75th percentile, the maximum, and the 95% confidence interval (CI) of the mean. Thickness ranges reflect manufacturer-reported categories.

Box-and-whisker plots show the distribution of measured hydrated volume (cc) across label-reported thickness ranges. Volume was measured after performing standardized hydration using the water displacement method. The box indicates the interquartile range (25th–75th percentiles), the center line indicates the median, whiskers extend to 1.5 times the interquartile range, and individual points denote outliers.

Two-way ANOVA was performed with the manufacturer and thickness range as fixed factors to evaluate the main effects of the manufacturer and thickness group, as well as their interaction, on the measured hydrated volume. The table reports sums of squares, degrees of freedom, F values, and *p*-values. Asterisks indicate statistical significance (*p* < 0.05).

One-way ANOVA demonstrated significant differences across thickness categories in measured hydrated volume (F = 86.05, *p* < 0.001) and calculated thickness (F = 77.29, *p* < 0.001), whereas the measured hydrated area did not differ significantly across thickness categories (F = 1.53, *p* = 0.208). In the shared thickness category of 1.0–2.0 mm, the measured hydrated volume was 85.8 cc for SCDerm^®^, 76.7 cc for MyDerm^®^, and 84.6 cc for CGDerm^®^. Although these values appeared to differ numerically among manufacturers, the difference was not statistically significant (F = 3.04, *p* = 0.052) (Supplementary Table S1, Figure 1).

One-way analysis of variance (ANOVA) was used to test differences across thickness ranges for the measured hydrated volume, measured hydrated area, and calculated thickness. Additional one-way ANOVA analyses compared manufacturers within the shared 1.0–2.0 mm thickness category. The table reports F statistics and corresponding *p*-values. Statistical significance was defined as *p* < 0.05.

Box-and-whisker plots depict the measured hydrated volume (cc) categorized by thickness range and manufacturer, as shown in Figure 1. The inset summarizes the testing differences in one-way ANOVA results across thickness ranges and, within the shared 1.0–2.0 mm category, differences across manufacturers.

### 3.2. Measured Hydrated ADM Area

**Figure 2:**
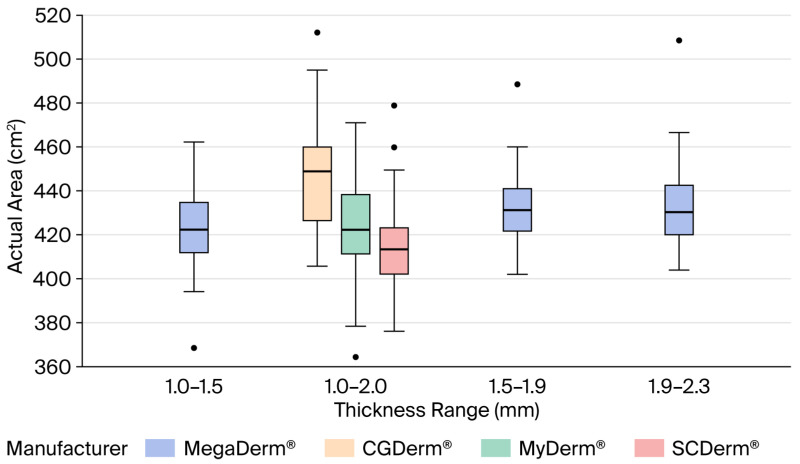
The measured hydrated area categorized by manufacturer and thickness range.

The estimated hydrated area and the measured hydrated area differed significantly across manufacturers, with all groups showing higher mean measured hydrated areas than the expected values (SCDerm^®^, 414.74 cm^2^; MyDerm^®^, 424.41 cm^2^; CGDerm^®^, 445.37 cm^2^; MegaDerm^®^, 422.98 cm^2^, 431.88 cm^2^, and 435.67 cm^2^; pooled MegaDerm^®^ mean, 428.06 cm^2^) (Table 4). Measured hydrated area values categorized according to manufacturer and thickness group are shown in Table 5 and Figure 2. One-way ANOVA across all thickness groups showed no significant difference in measured hydrated area (F = 1.53, *p* = 0.208). However, within the shared 1.0–2.0 mm thickness group, the measured area differed significantly among manufacturers (F = 14.98, *p* < 0.001) (Figure 2).

The mean estimated hydrated area was the label-reported area of a 20 × 20 cm sheet (400 cm^2^). The measured hydrated area was calculated from the post-hydration measured length and width of each sheet. Values are presented as mean ± SD. The mean difference represents the measured hydrated area minus the estimated hydrated area. *p*-values were obtained using one-sample *t* tests comparing the measured hydrated area with the mean estimated hydrated area within each manufacturer and thickness subgroup.

The measured hydrated area (cm^2^) was calculated from the post-hydration measured length and width of each ADM sheet. For each manufacturer and thickness subgroup, the table reports the mean, SD, minimum, 25th percentile, median, 75th percentile, maximum, and 95% CI of the mean. Thickness ranges reflect manufacturer-reported categories.

Box-and-whisker plots show the distribution of the measured hydrated area (cm^2^) across label-reported thickness ranges. The measured hydrated area was calculated from the post-hydration measured length and width of each sheet. Plot elements are as defined in Figure 1.

Box-and-whisker plots depict the measured hydrated area (cm^2^) categorized by thickness range and manufacturer, as shown in Figure 2. The inset summarizes one-way ANOVA results testing differences across thickness ranges and, within the shared 1.0–2.0 mm category, differences across manufacturers.

### 3.3. Post-Hydration Calculated Thickness

The calculated thickness (cc/cm^2^), defined as the hydrated volume divided by the actual measured area in the hydrated state, adequately reflected the characteristics of each manufacturer and thickness group (Table 6). In MegaDerm^®^, the calculated thickness showed a stepwise increase with increasing thickness group size, and differences were also observed among CGDerm^®^, MyDerm^®^, and SCDerm^®^ within the shared 1.0–2.0 mm thickness group. One-way ANOVA across all thickness groups demonstrated a significant difference in calculated thickness (F = 77.29, *p* < 0.001). Similarly, within the shared 1.0–2.0 mm thickness group, the calculated thickness differed significantly among manufacturers (F = 3.34, *p* = 0.039) (Table 6, Supplementary Table S1).

The calculated thickness was defined as the measured hydrated volume (cc) divided by the measured hydrated area (cm^2^), yielding a value measured in cc/cm^2^, which is dimensionally equivalent to centimeters. This variable was interpreted as the actual post-hydration thickness of ADM and converted to millimeters when appropriate. Values are presented as mean ± SD for each manufacturer and thickness subgroup.

### 3.4. Size- and Thickness-Specific Reference Table for Estimated Volume

Using the values derived from the measured 20 × 20 cm^2^ samples for each manufacturer and thickness subgroup, the estimated hydrated volumes were generated for various size–thickness combinations (Table 7). These values should be interpreted not as direct measurements but as derived reference estimates based on subgroup-specific mean calculated thickness. Extrapolation from the 20 × 20 cm^2^ samples to other sizes, including 16 × 16 cm^2^ and 18 × 18 cm^2^, was considered justifiable because all manufacturers stated, either through official statements or documentation, that differences in ADM size are attributable solely to trimming within the same manufacturing process. To assess the validity of the derived manufacturer-specific reference table, an additional comparison was performed using separate raw validation data. To avoid self-referential agreement with the 20 × 20 cm^2^ samples used in derivation, the validation analysis excluded the 20 × 20 cm^2^ data and included only the 18 × 18 cm^2^ data.

Reference volumes were derived to support volumetric planning when only the label-reported size and thickness range were available. For directly measured thickness subgroups, the subgroup mean calculated thickness (Table 6) was multiplied by the target area of each size (16 × 16 cm = 256 cm^2^; 18 × 18 cm = 324 cm^2^; 20 × 20 cm = 400 cm^2^) to obtain the estimated hydrated volume. SDs were propagated linearly with area under the assumption that the variation in the calculated thickness remained constant within each manufacturer and thickness subgroup. Values are presented as estimated mean ± SD (cc).

Derived estimates were generated as described in Table 7 using manufacturer- and thickness subgroup-specific calculated thickness values and target areas. For the additional 2.0–3.0 mm category, which was not directly measured in the 20 × 20 cm cohort for some manufacturers, the calculated thickness parameter was extrapolated from the corresponding 1.0–2.0 mm subgroup via scaling with the ratio of thickness-range midpoints (2.5/1.5). Values are presented as estimated mean ± SD (cc).

Direct comparison was possible in six subgroups. In all six, the raw mean measured hydrated volume showed overall agreement with the newly derived hydrated volumes. Four subgroups fell within the derived mean ± 1 standard deviation (SD), and the remaining two fell within the ± 2 SD range. Specifically, agreement within ± 1 SD was observed for CGDerm^®^ 18 × 18 cm^2^, 1.0–2.0 mm; MyDerm^®^ 18 × 18 cm^2^, 1.0–2.0 mm; and SCDerm^®^ 18 × 18 cm^2^, 1.0–2.0 mm. MegaDerm^®^ 18 × 18 cm^2^, 1.0–1.5 mm, fell outside the ±1 SD range but remained within the ±2 SD range. (Table 8).

To assess concordance between the derived hydrated volumes and directly measured data not used for derivation, the table compares the estimated mean ± SD (cc) for 18 × 18 cm sheets with the raw measured hydrated volume (cc) from an independent validation dataset. The assessment field indicates whether the raw mean fell within ±1 SD (high concordance) or within ±2 SD (moderate concordance) of the estimated value.

These findings suggest that the newly derived hydrated volume table is generally concordant with directly comparable raw validation data not used for derivation. Therefore, the proposed reference table may serve as a practical derived estimate for use in real-world clinical planning.

## 4. Discussion

Various methods exist for measuring acellular dermal matrix (ADM) volume. First, the fluid displacement method is a classical yet reliable approach that measures the true physical volume of the graft. Second, the digital micrometer or grid-point method utilizes a constant-force ratchet stop device to perform precise grid calculations. Lastly, computed tomography (CT) or magnetic resonance imaging (MRI)-based volumetric analysis offers a non-invasive, highly accurate three-dimensional evaluation [18]. This method utilizes Digital Imaging and Communications in Medicine (DICOM) software to perform three-dimensional (3D) reconstruction and voxel-based segmentations, allowing for the precise measurement of both pre- and postoperative matrix volumes. Among the various volumetric techniques, the fluid displacement method was chosen for this study due to its intraoperative feasibility, high inter-institutional reproducibility, and operational simplicity. This approach provides a significant advantage, as the volume can be immediately confirmed intraoperatively prior to in vivo placement.

This study was meaningful as it quantitatively characterized the post-hydration volumetric properties of ADM used in prepectoral breast reconstruction according to manufacturer and thickness group and, on this basis, proposed a manufacturer-specific reference table. The measured hydrated volume differed substantially according to thickness range, whereas variation in the measured hydrated area was relatively limited. Moreover, products with the same manufacturer-reported size and thickness range did not show identical measured hydrated volume, area, or calculated thickness across manufacturers. These findings indicate that ADM functions not only as a material for implant coverage but also as a structural component with a measurable volumetric contribution [13,16,19]. Consequently, ADM selection may affect implant pocket geometry, superior pole fullness, breast contour, and symmetry [15,17,20], and its hydrated characteristics should be considered together with label information during surgical planning.

Furthermore, the derived hydrated volume values presented in this study are not values directly measured for every product size but practical estimates derived from the hydrated volume and area measured in the 20 × 20 cm^2^ products. These estimates, however, were not based on assumption alone. In communications with each manufacturer, all consistently stated that products from the same manufacturer undergo the same manufacturing process and that differences among sizes are attributable only to trimming. This information provided the basis for deriving reference values for other sizes. Nevertheless, a perfectly proportional relationship cannot be assumed across all sizes and conditions in clinical practice, and possible variation according to donor harvest site should also be considered. For example, for MegaDerm^®^, the donor site is specified, and differences in density may exist according to donor origin in the order of back, abdomen, and thigh. Thus, even within products from the same manufacturer, a certain degree of biological heterogeneity may be present [21].

Another consideration is that although, in this study, we focused on hydrated volumetric characteristics, other material properties were not directly assessed. In actual surgical practice, some products may be perceived as relatively more elastic or softer than others, and these handling characteristics may affect draping behavior, pocket adaptation, tension distribution, and even the surgeon’s subjective impression of thickness. Thus, inter-product differences not fully explained by measured hydrated volume or calculated thickness alone may exist. However, because such properties were not objectively validated with biomechanical parameters in this study, they should be interpreted as clinical observations rather than experimentally established findings.

Furthermore, a critical methodological assumption in this study involves the generation of the reference values for the 2.0–3.0 mm thickness subgroups of MyDerm, CGDerm, and SCDerm. Due to the lack of direct intraoperative data for these specific thicker variants within our dataset, these reference parameters were calculated via linear-scaling extrapolation, applying a midpoint ratio of 2.5/1.5 to the empirical data obtained from the 1.0–2.0 mm subgroups. Clinicians must interpret these specific rows with caution, as this mathematical projection inherently assumes a perfectly uniform, linear increase in volumetric properties across varying manufacturer specifications. Because this linear-scaling assumption lacks direct empirical validation within our current patient cohort, it introduces a degree of statistical uncertainty. In clinical reality, thicker dermal matrices may exhibit non-linear fluid dynamics, altered swelling ratios, or distinct microstructural behaviors during hydration that diverge from their thinner counterparts. Consequently, these extrapolated values should be utilized strictly as theoretical planning aids rather than validated biological constants.

This study has several limitations. First, as a single-center retrospective analysis, the study population and product selection may have been influenced by institution-specific experience, and the findings should, therefore, be generalized with caution. Second, direct measurements were limited to the 20 × 20 cm^2^ products, and external validation of the derived reference values for other sizes remains necessary. Third, because potentially relevant determinants of inter-product variation, including donor site, matrix structure, hydration behavior, and processing method, were not directly investigated, this study should be interpreted as providing quantitative evidence that such differences exist rather than as clarifying their precise causes. Fourth, the fluid displacement method itself carries inherent technical limitations; the extra intraoperative handling introduces a theoretical risk to strict sterility, and the visual meniscus readings can be sensitive to the subjective degree of surface blotting. Furthermore, this ex vivo measurement reflects the matrix in an uncompressed state, which may not fully replicate the actual in vivo volume under mechanical tension after pocket closure. Accordingly, the proposed reference table should be understood not as an absolute standard for definitively predicting the actual hydrated volume of an individual product but as a practical planning aid. Future investigations incorporating multicenter datasets, external validation cohorts, and biomechanical property assessment may allow for a more precise understanding of differences among ADM products.

## 5. Conclusions

ADM used in prepectoral breast reconstruction exhibits distinct post-hydration volumetric characteristics according to the manufacturer and thickness group. These differences are attributable primarily to variation in calculated thickness, or volume per unit area, rather than to differences in the measured hydrated area alone. The measured hydrated volume and calculated thickness differed significantly across thickness ranges, and manufacturer-specific differences were observed within the shared thickness group. The manufacturer-specific derived reference table presented in this study should be understood not as a definitive substitute for direct measurement but as a practical reference estimate for performing volumetric planning in prepectoral breast reconstruction. Its use may help reduce error in preoperative implant volume planning and improve postoperative symmetry.

## Figures and Tables

**Table 1:** The specifications and sample distribution of acellular dermal matrices (ADMs) included in the direct measurement cohort.

Manufacturer	Total (%)	*N*	Thickness (mm)
CGDerm^®^	35 (17.5)	35	1.0–2.0
MegaDerm^®^	87 (43.5)	45	1.0–1.5
		24	1.5–1.9
		18	1.9–2.3
MyDerm^®^	48 (24.0)	48	1.0–2.0
SCDerm^®^	30 (15.0)	30	1.0–2.0
Total	200 (100)	200	

**Table 2:** A comparison of the estimated hydrated volume and measured hydrated volume for 20 × 20 cm ADM sheets.

Manufacturer	*p*-Value	T	Mean Difference	Measured Hydrated Volume (cc)	Estimated Hydrated Volume (cc)	*n*	Thickness (mm)
CGDerm^®^	<0.001	7.79	24.63	84.63 ± 18.70	60.00	35	1.0–2.0
MegaDerm^®^	<0.001	7.43	18.20	68.20 ± 16.44	50.00	45	1.0–1.5
MegaDerm^®^	<0.001	14.48	43.50	111.50 ± 14.72	68.00	24	1.5–1.9
MegaDerm^®^	<0.001	9.97	56.67	140.67 ± 24.11	84.00	18	1.9–2.3
MyDerm^®^	<0.001	5.93	16.75	76.75 ± 19.56	60.00	48	1.0–2.0
SCDerm^®^	<0.001	9.69	25.87	85.87 ± 14.62	60.00	30	1.0–2.0

**Table 3:** The descriptive statistics of measured hydrated ADM volume categorized by manufacturer and thickness subgroup.

Manufacturer	95% CI Upper	95% CI Lower	Max (cc)	75th % (cc)	Median (cc)	25th % (cc)	Min. (cc)	Shapiro–Wilk *p*	SD (cc)	Mean (cc)	Thickness Range
CGDerm^®^	90.8	78.4	120	91.5	83	72	52	0.056	18.7	84.6	1.0–2.0
MegaDerm^®^	73.0	63.3	115	82	63	56	43	0.015	16.4	68.2	1.0–1.5
	117.3	105.6	137	121	111	104.5	70	0.470	14.7	111.5	1.5–1.9
	151.8	129.5	185	156.7	132.5	124	108	0.060	24.1	140.6	1.9–2.3
MyDerm^®^	82.2	71.2	150	83	75	68	42	0.001	19.5	76.7	1.0–2.0
SCDerm^®^	91.0	80.6	112	92.75	85	79.25	54	0.439	14.6	85.8	1.0–2.0

**Table 4:** A comparison of the estimated hydrated area and measured hydrated area for 20 × 20 cm ADM sheets.

Manufacturer	*p*-Value	T	Mean Difference	Measured Hydrated Area (cm^2^)	Estimated Hydrated Area (cm^2^)	*N*	Thickness (mm)
CGDerm^®^	<0.001	10.31	45.37	445.37 ± 26.03	400.00	35	1.0–2.0
MegaDerm^®^	<0.001	8.42	22.98	422.98 ± 18.31	400.00	45	1.0–1.5
MegaDerm^®^	<0.001	8.30	31.88	431.88 ± 18.82	400.00	24	1.5–1.9
MegaDerm^®^	<0.001	6.35	35.67	435.67 ± 23.85	400.00	18	1.9–2.3
MyDerm^®^	<0.001	7.67	24.41	424.41 ± 22.05	400.00	48	1.0–2.0
SCDerm^®^	0.001	3.61	14.74	414.74 ± 22.35	400.00	30	1.0–2.0

**Table 5:** The descriptive statistics of the measured hydrated area categorized by manufacturer and thickness subgroup.

Manufacturer	95% CI Upper	95% CI Lower	Max (cm^2^)	75th % (cm^2^)	Median (cm^2^)	25th % (cm^2^)	Min. (cm^2^)	SD (cm^2^)	Mean (cm^2^)	Thickness Range
CGDerm^®^	453.9	436.7	512.3	459.9	448.6	426.4	405.6	26.0	445.3	1.0–2.0
MegaDerm^®^	428.5	417.6	462.3	435.2	423.3	412.0	368.6	18.4	423.3	1.0–1.5
	439.4	424.3	488.4	440.9	429.4	421.8	402.0	18.8	431.9	1.5–1.9
	446.6	424.6	508.5	442.5	430.5	420.2	404.0	23.8	435.7	1.9–2.3
MyDerm^®^	430.6	418.1	470.9	438.1	422.0	411.5	364.7	22.0	424.4	1.0–2.0
SCDerm^®^	422.7	406.7	479.0	423.1	413.0	401.9	376.2	22.3	414.7	1.0–2.0

**Table 6:** The calculated thickness categorized by manufacturer and thickness subgroup.

Manufacturer (ADM)	SD (cc/cm^2^)	Mean Calculated Thickness (mm)	Label-Reported Thickness (mm)
CGDerm^®^	0.05	2.11	1.0–2.0
MegaDerm^®^	0.04	1.70	1.0–1.5
	0.04	2.83	1.5–1.9
	0.05	3.51	1.9–2.3
MyDerm^®^	0.04	1.91	1.0–2.0
SCDerm^®^	0.03	2.14	1.0–2.0

**Table 7:** The derived hydrated ADM volume reference estimates categorized by size and thickness for MegaDerm, MyDerm, CGDerm and SCDerm.

Manufacturer (ADM)	SD (cc)	Mean (cc)	Thickness (mm)	Size (cm^2^)
MegaDerm^®^	10.5	43.6	1.0–1.5	16 × 16
	9.4	71.4	1.5–1.9	16 × 16
	15.4	90.0	1.9–2.3	16 × 16
	13.3	55.2	1.0–1.5	18 × 18
	11.9	90.3	1.5–1.9	18 × 18
	19.5	113.9	1.9–2.3	18 × 18
	16.4	68.2	1.0–1.5	20 × 20
	14.7	111.5	1.5–1.9	20 × 20
	24.1	140.7	1.9–2.3	20 × 20
MyDerm^®^	15.8	62.2	1.0–2.0	18 × 18
	26.4	103.6	2.0–3.0	18 × 18
	19.6	76.8	1.0–2.0	20 × 20
	32.6	127.9	2.0–3.0	20 × 20
CGDerm^®^	15.2	68.5	1.0–2.0	18 × 18
	25.3	114.2	2.0–3.0	18 × 18
	18.7	84.6	1.0–2.0	20 × 20
	31.2	141.0	2.0–3.0	20 × 20
SCDerm^®^	11.8	69.6	1.0–2.0	18 × 18
	19.7	115.9	2.0–3.0	18 × 18
	14.6	85.9	1.0–2.0	20 × 20
	24.4	143.1	2.0–3.0	20 × 20

**Table 8:** The validation of the derived hydrated volume reference table using independent 18 × 18 cm ADM data.

Manufacturer	Raw Mean (cc)	Derived Mean ± SD (cc)	Thickness	Mean Percent Difference (%)	95% CI Upper (%)	95% CI Lower (%)	Size	N
CGDerm^®^	78.1	68.5 ± 15.2	1.0–2.0	+14.0	+21.9	+6.0	18 × 18	16
MegaDerm^®^	76.3	55.2 ± 13.3	1.0–1.5	+38.2	+68.7	+7.7	18 × 18	7
MyDerm^®^	65.0	62.2 ± 15.8	1.0–2.0	+4.5	+14.2	−5.2	18 × 18	24
SCDerm^®^	64.5	69.6 ± 11.8	1.0–2.0	−7.3	−180.8	+166.1	18 × 18	2

## Data Availability

The data presented in this study are available on request from the corresponding author. The data are not publicly available due to consideration for the patients’ privacy.

## Supplementary Materials

The following supporting information can be downloaded at: https://www.mdpi.com/article/10.3390/jcm15155948/s1.
