# Supplementary material for: Actual Hydrated Volume of Acellular Dermal Matrix in Prepectoral Breast Reconstruction
Source: J Clin Med. 2026 Jul 30;15(15):5948. doi: 10.3390/jcm15155948 (PMC13467060; doi:10.3390/jcm15155948)
Supplement: Supplementary file 1 [file jcm-15-05948-s001.zip › jcm-4399485-supplementary.pdf]

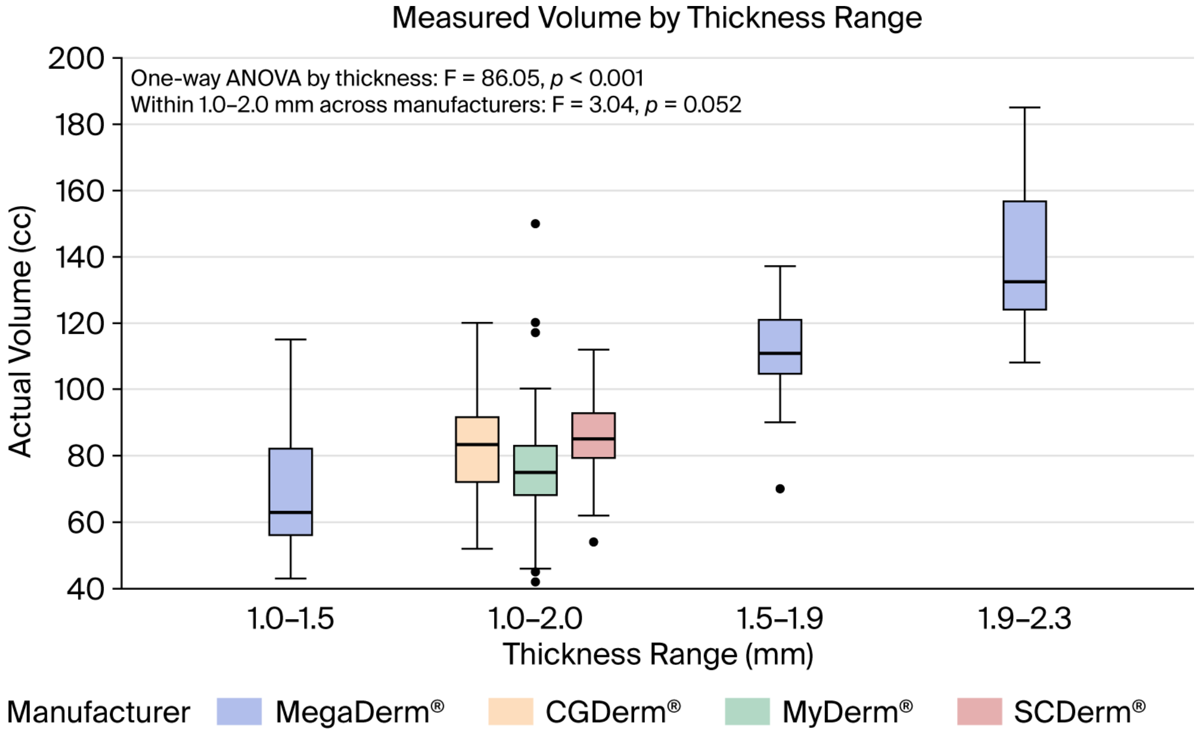

Figure S1. The measured hydrated volume categorized by thickness range with ANOVA results.

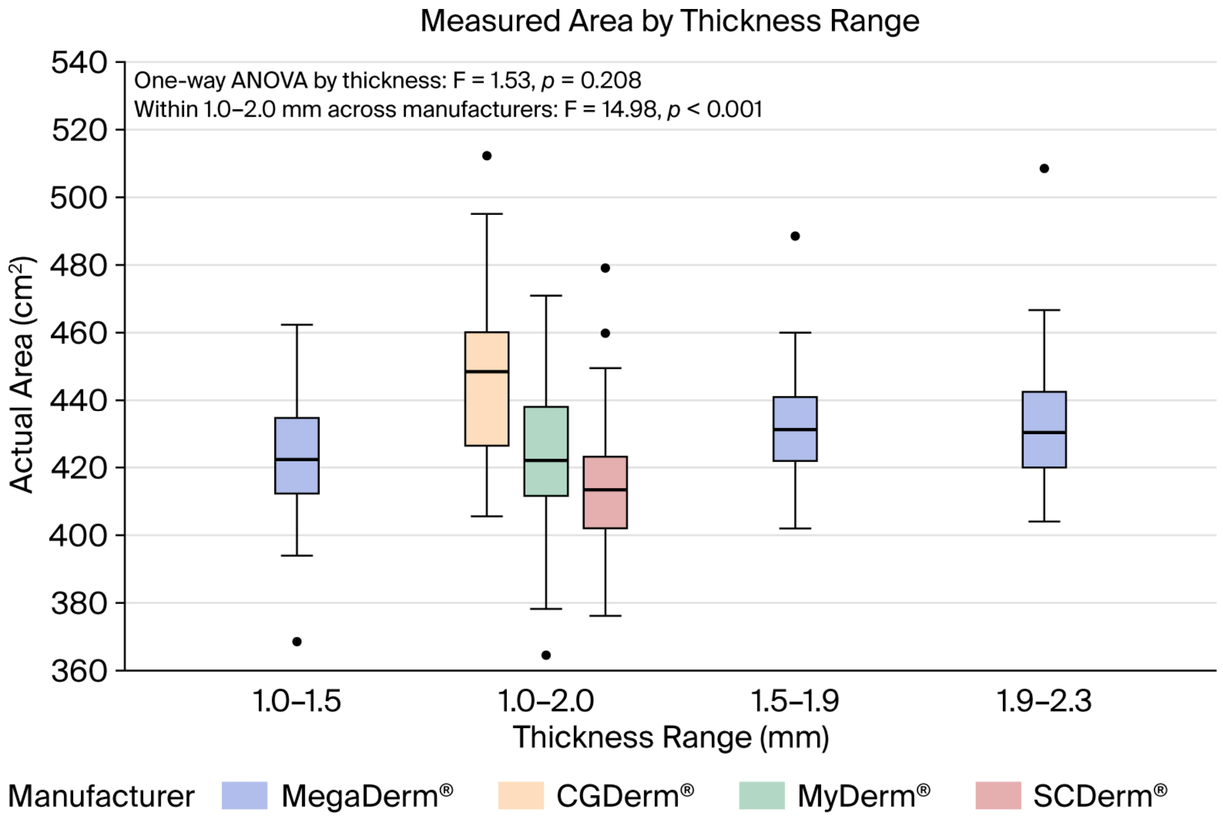

Figure S2. The measured hydrated area categorized by thickness range with ANOVA results.

Table S1. A summary of one-way ANOVA comparisons for the measured hydrated volume, measured hydrated area, and calculated thickness.

***P*-Value**	**F-Value**	**df**	**Sum of Squares**	**Source of Variation**
<0.001 *	33.67	3	32,709.60	Manufacturer
<0.001 *	123.58	3	120,067.90	Thickness
<0.001 *	60.51	9	176,371.60	Interaction

Table S2. Two-way ANOVA of the measured hydrated volume categorized by manufacturer and thickness range.

**Interpretation**	***P*-Value**	**F**	**Test**	**Comparison**
Measured hydrated volume increased significantly across thickness categories.	<0.001	86.05	One-way ANOVA	Measured volume across thickness ranges
Measured hydrated area did not differ significantly across thickness categories.	0.208	1.53	One-way ANOVA	Measured area across thickness ranges
Calculated thickness (cc/cm^2) closely reflected thickness categories.	<0.001	77.29	One-way ANOVA	Calculated density across thickness ranges
Inter-manufacturer difference in volume was not statistically significant.	0.052	3.04	One-way ANOVA	Volume within the 1.0–2.0 mm group across manufacturers
Measured hydrated area differed significantly among manufacturers within the shared thickness group.	<0.001	14.98	One-way ANOVA	Area within the 1.0–2.0 mm group across manufacturers
Calculated thickness differed significantly among manufacturers within the shared thickness group.	0.039	3.34	One-way ANOVA	Density within the 1.0–2.0 mm group across manufacturers
